# Comparing Beers, STOPP and MALPIP criteria in detecting potentially inappropriate medications, clinical outcomes, and cost impacts among older Malaysians: a cohort study

**DOI:** 10.1080/20523211.2024.2436896

**Published:** 2024-12-18

**Authors:** Chee Tao Chang, Huan-Keat Chan, Aie Yen Tan, Siti Fatimah Kamis, Yee Ling Yeo, Muhammad Azuan Azman, Shamini Rama, James Yau Hon Voo, Hoo Seng Tan, Janice Kah Weng Kwan, Xin Yi Ooi, Philip Rajan, Siew Li Teoh, Shaun Wen Huey Lee

**Affiliations:** a School of Pharmacy, Monash University Malaysia, Subang Jaya, Malaysia; b Clinical Research Centre Hospital Raja Permaisuri Bainun, Institute for Clinical Research, National Institute of Health, Ministry of Health Malaysia, Ipoh, Malaysia; c Clinical Research Centre Hospital Sultanah Bahiyah, Institute for Clinical Research, National Institute of Health, Ministry of Health Malaysia, Alor Setar, Malaysia; d Pharmacy Department, Hospital Sultan Ismail, Ministry of Health Malaysia, Johor Bharu, Malaysia; e Pharmacy Department, Hospital Raja Permaisuri Bainun, Ministry of Health Malaysia, Ipoh, Malaysia; f Pharmacy Department, Hospital Bahagia Ulu Kinta, Ministry of Health Malaysia, Tanjung Rambutan, Malaysia; g Pharmacy Department, Hospital Duchess of Kent, Ministry of Health Malaysia, Sandakan, Malaysia

**Keywords:** Aged, potentially inappropriate medications, cost savings, treatment outcome

## Abstract

**Background:** Potentially inappropriate medications (PIMs) are associated with adverse outcomes and higher healthcare costs in older adults. Explicit screening criteria like the Beers Criteria, STOPP criteria, and the Malaysian Potentially Inappropriate Prescribing (MALPIP) criteria served to identify PIMs, but comparative data are scarce.

**Aim:** To evaluate the prevalence of PIMs identified by Beers 2019, STOPP version 2 and MALPIP criteria in Malaysian older adults and examine their predictive ability for adverse outcomes and cost-saving potential.

M**ethods:** A historical cohort study was conducted among older adults aged ≥ 60 years on five or more medications in four Malaysian tertiary hospitals. PIMs were identified using Beers, STOPP, and MALPIP criteria. Sensitivity, specificity and predictive abilities of these criteria were analysed against clinical outcomes. Monthly cost savings were calculated based on hypothetical deprescribing scenarios.

**Results:** Among 1069 patients, the prevalence of PIMs was 89.1% using MALPIP, 51.3% with Beers, and 37.0% with STOPP criteria. A moderate concordance was seen between Beers and STOPP criteria (κ =  0.437), and the lowest agreement was observed between the STOPP and MALPIP (κ =  0.131). STOPP criteria significantly predicted hospital readmissions (*p* = 0.003), while Beers and MALPIP did not show significant predictive abilities across all outcomes. The most common PIMs identified were proton pump inhibitors (PPIs) and nonsteroidal anti-inflammatory drugs (NSAIDs). Deprescribing scenarios based on these criteria indicated potential monthly cost savings of MYR 4.83 to MYR 44.84 per patient, with the greatest savings associated with MALPIP criteria.

**Conclusion:** MALPIP demonstrated the highest potential for cost savings, the highest sensitivity but the lowest specificity in PIM detection. Context-specific assessments and clinical judgment are crucial in optimising medication safety and efficacy in geriatric pharmacotherapy. Further research is needed to refine PIM criteria to better predict clinical outcomes and balance the benefits and risks of deprescribing in diverse healthcare settings.

## Introduction

The global population is aging at an unprecedented rate, with an estimated 2.1 billion older adults aged 60 years by 2050 (Jones & Dolsten, 2024). As a consequent, an increase in the prevalence of chronic diseases is expected (Ansah & Chiu, 2022; Hacker, 2024). Polypharmacy, often defined as the use of five or more medications is expected (Varghese et al., 2024). Studies have suggested that polypharmacy can be harmful as it is associated with a higher risk of experiencing an adverse drug reaction (Osanlou et al., 2022), fall (S. AlHarkan et al., 2023) and drug–drug interactions (Hermann et al., 2021).

To address this, deprescribing or the removal of medications where the harms outweigh the benefits especially when safer alternatives are available are recommended. These medications are often known as potentially inappropriate medications (PIMs). PIMs are very prevalent in the society, with a prevalence ranging from 23.6% in Oceania to as high as 47% in Africa (Tian et al., 2023). In Malaysia, the pooled prevalence of polypharmacy and PIMs were estimated at 49.5% and 28.9% respectively (C.-T. Chang et al., 2021). As such, identifying and minimising the use of PIMs is crucial for enhancing patient safety and reducing healthcare costs (Kua et al., 2021; Okafor et al., 2024).

To aid in deprescribing, several guidelines and recommendations have been developed to identify PIMs. These include the American Geriatrics Society (AGS) Beers Criteria (By the, 2019 American Geriatrics Society Beers Criteria® Update Expert Panel, 2019), originally developed by Mark H. Beers in 1991, regularly updated by the American Geriatrics Society (AGS) to provide guidance regarding medications that should be avoided; the Screening Tool of Older Persons’ Prescriptions (STOPP) criteria initially developed in 2008, with second version published in 2015, a physiological systems-based explicit set of criteria that relates clinically important prescribing problems and PIMs (O’Mahony et al., 2015); and the Malaysian potentially inappropriate prescribing (MALPIP) criteria, a Malaysian PIM screening tool in older adults with three sections and 169 items (C. T. Chang et al., 2023).

Several randomised controlled studies have assessed the impact of deprescribing utilising the explicit PIM screening criteria described above with varying results (Omuya et al., 2023). For instance, Cateau and colleagues evaluated the effects of a deprescribing intervention in Swiss nursing homes using the STOPP criteria. The intervention led to a significant reduction in the overall number of medications and doses of PIMs (Cateau et al., 2021). Another study which implemented medication reviews among older residents in nursing homes using both Beers and STOPP criteria found that deprescribing reduced hospitalisation and mortality rates but also resulted in a daily cost saving of US$ 11.42 (Kua et al., 2021).

Despite the availability of these tools, there is limited evidence on their comparative effectiveness and predictability of using PIMs for adverse outcomes especially in Asia, where the ageing population is expected to increase exponentially. The current study aims to fill this gap by evaluating the prevalence of PIMs detected by the 2019 Beers, STOPP version 2, and MALPIP criteria in a cohort of older adults receiving outpatient care in tertiary hospitals in Malaysia, as well as deprescribing preferences through case scenario. Additionally, the study examines the predictive validity of these three criteria against adverse clinical outcomes, including emergency department (ED) visits, hospital readmissions, and mortality.

## Methods

This cohort study included older adults aged ≥ 60 years old who were on five or more medications receiving medical care in four tertiary hospitals in the states of Perak, Johor, and Sabah from January 2019 to September 2021. Prescriptions with incomplete data, such as missing information on patient age, diagnosis, or comorbidities, were excluded from the study. A consecutive sampling method was employed. Index prescription was defined as the first date that met all the inclusion criteria, with subjects retrospectively followed for an 18-month period starting from the date of the index prescription. The study received ethics approval from the Medical Research & Ethics Committee (21-01952-GM8).

### Sample size

Based on a previous study (C.-T. Chang et al., 2021), the prevalence of PIMs among elderly patients was 28.9%. With an alpha risk of 0.03 and a confidence interval of 95%, it was estimated that 841 patients would be needed to detect an adequate number of PIM cases (Raosoft sample size calculator). Considering an anticipated 20% rate of incomplete data, a minimum total of 1,050 patients was required. A total of 1,069 samples were successfully obtained, achieving 101.8% of the intended sample size.

### Data collection

All patient-related information including medication history, comorbidities and admission history were obtained by trained pharmacists through the hospital's health information system or medical records. These include the all-cause emergency department (ED) readmissions, all-cause readmissions, and mortality, which were cross-verified with the National Registry Department at 18 months. Patient’s medication data were then coded using the Anatomical and Therapeutic Classification (ATC) system. PIM was classified using three tools: (a) the MALPIP criteria, (b) the 2019 Beers criteria, and (c) the STOPP version 2 criteria.

### Statistical analysis

Results were presented as frequencies and percentages for categorical variables and as means (SD) for numerical variables. The sensitivity and specificity of the three different PIM identification criteria against emergency visits, readmission, and mortality outcomes were measured, based on whether the subjects experienced the clinical outcome, and whether the PIM criteria yielded a positive or a negative result.

The Cohen’s Kappa inter-rater reliability coefficient between the three criteria was generated using crosstabs, which 0.01–0.20 was defined as none to slight agreement, 0.21–0.40 as fair, 0.41–0.60 as moderate, 0.61–0.80 as substantial, and 0.81–1.00 as almost perfect agreement. Univariate and multivariate logistic regression analysis were performed to determine the association between the patient-related factors, three different PIM identification criteria against emergency visits, readmission, and mortality outcomes.

Subsequently, ten cases were randomly selected to assess the specificity of the PIM criteria. Three geriatricians involved in the development of the MALPIP criteria were invited to participate in the review process. Each case was presented to the geriatricians, including detailed information such as patient age, gender, medical history, admission history, current medication list, and PIMs identified based on the STOPP, Beers, and MALPIP criteria (Table 1). Each geriatrician independently reviewed the cases and was asked to determine whether they would discontinue the identified PIMs if faced with similar cases in their clinical practice. The cost of medications was obtained from the local procurement inventory database, and potential monthly cost savings were calculated assuming that PIMs were deprescribed according to the three different criteria, based on three hypothetical scenario, i.e. stopping all, 50% or 25% of the PIM. All statistical analyses were performed using Statistical Package for Social Sciences (IBM® SPSS® Statistics version 25).

**Table 1.** Comparison of Beers, STOPP and MALPIP criteria.

	Beers	STOPP	MALPIP
Classification	Drug class	Physiological system	Drug class
Disease-specific PIM	Yes	Yes	Yes
Potential Prescribing omission	No	Yes	Yes
Drug–drug interactions	Yes	Yes	No
Quality of evidence	Yes	No	No
Strength of recommendations	Yes	No	No
Version used	2019	2015	2023

## Results

Of a total of 1093 patient prescription identified, 24 were excluded due to missing data and 1,069 were included in the current study. The majority of participants (589, 55.1%) were aged between 60 and 70 years, and most (888, 83.1%) were taking between 5 and 10 medications.

### Classes of PIM detected using Beers, STOPP and MALPIP

Over the entire post-index period, we found that most patients were exposed to at least 1 PIM, with the highest number of PIMs detected using the MALPIP criteria (*n* = 952, 89.1%) compared to 51.3% using Beers criteria (*n* = 548) or the STOPP criteria (*n* = 395, 37.0%) This included 321 patients (30.0%) who were identified as having PIMs by all three criteria (Table 2).

**Table 2.** Demographic characteristics, number of medications and PIMs detected among older adults studied (*n* = 1069).

Characteristics	Results (Number, %)
Age, median (IQR) Age Range	69.00 (10.0), 60–95
60–70	589 (55.1)
71–80	364 (34.1)
81 and above	116 (10.9)
Gender	
Male	536 (50.1)
Female	533 (49.9)
Mean (median) comorbid per subject	2.93 ± 1.27, Median = 3.00
Average number (median) of medications	8.12 ± 3.19, Median = 7.00
Hospitalization in the past 12 months	
Yes	238 (22.3)
No	831 (77.7)
Total number of medications	
5–10	888 (83.1)
11–15	145 (13.6)
16–20	30 (2.8)
> 20	6 (0.6)
Detected at least one PIM by STOPP	395 (37.0)
1	304 (28.4)
2	75 (7.0)
3	13 (1.2)
4	3 (0.3)
Mean (SD)	1.28 ± 0.56
Detected at least one PIM by Beers	548 (51.3)
1	392 (36.7)
2	125 (11.7)
3	28 (2.6)
4	3 (0.5)
Mean (SD)	1.35 ± 0.60
At least one PIM by MALPIP	952 (89.1)
1	319 (29.8)
2	309 (28.9)
3	218 (20.4)
4	72 (6.7)
5 and above	34 (3.2)
Mean (SD)	2.16 ± 1.12
Did patient visit ED in the next 18 months	
Yes	266 (24.9)
No	803 (75.1)
ED visits, Mean (SD)	1.89 (1.47)
Did patient readmit in the next 18 months	
Yes	264 (24.7)
No	805 (75.3)
Readmission, Mean (SD)	1.79 (1.52)
Did patient survive 18 months later?	
Yes	953 (89.1)
No	116 (10.9)

The top 10 individual PIM for each criterion had many similar medications, but with some variations in the medication groups (Table 3). For instance, the STOPP criteria identified NSAIDs (84/518, 16.2%), antihistamines (75/518, 14.5%) and PPIs (71/518, 13.7%) as the top three classes. In contrast, the Beers criteria highlighted PPIs (212/760, 27.9%), opiate analgesics (122/760, 16.1%), and NSAIDs (83/760, 10.9%). Meanwhile, the MALPIP criteria detected PPIs (252/2085, 12.1%), diuretics (237/2085, 11.4%), and beta blockers (149/2085, 7.1%) as the three most frequently prescribed classes of inappropriate medications. Assuming that all PIMs were discontinued, there was a potential cost savings between MYR 4.83 to MYR 44.84 per patient, depending on criterion used (Table 4).

**Table 3.** Most commonly prescribed potentially inappropriate medication in 1069 older adults who visited outpatient pharmacy.

No.	STOPP		Beers		MALPIP	
	medications	Total	medications	Total	medications	Total
1.	Anti-inflammatory (NSAIDs)	84	Proton Pump Inhibitors	212	Proton Pump Inhibitors	252
2.	Antihistamines	75	Opiate analgesics	122	Diuretics	237
3.	Proton Pump Inhibitors	71	Anti-inflammatory (NSAIDs)	83	Beta Blockers	149
4.	Benzodiazepines	59	Alpha Blockers	65	Anti-inflammatory (NSAIDs)	147
5.	Opiate analgesics	33	Benzodiazepines	60	Opiate analgesics	138
6.	Diuretics	28	Antipsychotics	57	Immunosuppressants	135
7.	Beta Blockers	19	Antidepressants	52	Corticosteroids	115
8.	Antiparkinson	16	Antihistamines	32	Alpha Blockers	103
9.	Antidepressants	14	Diuretics	10	Antihistamines	86
10.	Antigout	10	Antiepileptic	10	Anti-arthritis	66
	Sum of top ten classes	409		703		1428
	Total medications detected	518		760		2085

**Table 4.** Monthly cost saving in three hypothetical scenarios when PIM was discontinued.

	Monthly cost savings if discontinued	STOPP (MYR)	Beers (MYR)	MALPIP (MYR)
Scenario A	Total cost savings, stop all PIM	7,733.16	15,184.55	43,450.23
	Average savings per patient	19.33	27.10	44.84
Scenario B	Total cost savings Stop 50% PIM	38,66.58	7,592.28	21,725.12
	Average savings per patient	9.66	13.53	22.42
Scenario C	Total cost savings Stop 25% PIM	1,933.29	3,796.14	10,862.56
	Average savings per patient	4.83	6.77	11.21

There was slight to moderate concordance among all three tools. A moderate concordance was seen between Beers and STOPP criteria (κ =  0.437), with a fair concordance between Beers and MALPIP (κ = 0.225). The lowest agreement was observed between the STOPP and MALPIP criteria, with a kappa value of 0.131.

### Sensitivity and specificity of STOPP, Beers and MALPIP and associations with emergency visits, hospitalisation and mortality

Nearly one quarter (266, 24.9%) of these patients required an emergency department visit post-index. Another 264 (24.7%) were readmitted to the hospital during the 18-month study period. Of the 1069 subjects included in this study, mortality occurred in 116 (10.9%) subjects.

The MALPIP criteria reported the highest level of sensitivity, but the lowest level of specificity in predicting the ED visits, readmissions and mortality. The Beers criteria had a moderate level of sensitivity and specificity, while the STOPP criteria reported the lowest level of sensitivity and the highest level of specificity (Table 5).

**Table 5.** Sensitivity and specificity of PIM criteria predicting clinical outcomes.

Criteria and outcomes	Sensitivity	Specificity
STOPP		
ED visits	35.3%	62.5%
Readmissions	29.9%	60.7%
Mortality	39.7%	63.4%
Beers		
ED visits	54.1%	49.7%
Readmissions	53.0%	49.3%
Mortality	56.9%	49.4%
MALPIP		
ED visits	87.6%	10.5%
Readmissions	87.5%	10.4%
Mortality	92.2%	11.3%

Individuals with a higher number of comorbidities, or had a previous hospitalisation history 12 months ago were more likely to require an emergency department visits, hospital readmissions, and mortality. Nevertheless only individuals identified with PIMs using the STOPP criteria were associated with an increased risk of readmission. Both Beers and MALPIP criteria did not significantly predict any clinical outcomes (Supplemental Material – Tables 1–3).

### Clinician deprescribing preferences

To examine the applicability of each criteria in clinical practice, we randomly identified 10 prescriptions which were shared with geriatricians who were asked to decide on which PIM which they would deprescribe. From the 92 medications used, 36 PIMs were detected, of which the geriatricians recommended stopping 16 (44.4%) of them in 7 patients. STOPP criteria only identified 6 PIMs, and 5 were recommended to deprescribe (83.3%). The Beers criteria detected 15 PIMs, with 9 (60.0%) recommended for deprescribing while MALPIP criteria flagged all 36 PIMs, of which 16 (44.4%) were subsequently recommended to stop by geriatricians.

## Discussion

To the best of our knowledge, this is the first study to compare the prevalence of PIMs using three distinct criteria, i.e. MALPIP, Beers 2019, and STOPP version 2 among older adults receiving outpatient care at tertiary hospitals in Malaysia. The three criteria consistently detected a high prevalence of PIMs, with each criterion identifying different types of inappropriate medications. The MALPIP criteria emerged as the most sensitive tool for detecting PIMs compared to Beers 2019 and STOPP version 2, although it exhibited the lowest specificity. Additionally, there was poor concordance between the three criteria.

Several studies have assessed the prevalence of PIMs among older adults in outpatient pharmacy settings. A study in Saudi Arabia reported that 57.6% of older adults treated in outpatient care were prescribed at least one PIM using the 2015 Beers criteria, aligning closely with our findings (Alhawassi et al., 2019). In contrast, a Malaysian study reported a lower prevalence of 39.4% among older adults in a tertiary hospital outpatient pharmacy (Ang et al., 2021). A large-scale systematic review, including 94 studies across 17 countries, found a global PIM prevalence of 36.7% in outpatient settings (Tian et al., 2023), which was lower than those identified using the Beers and MALPIP criteria, but comparable to the STOPP criteria in this study.

While this study found a high prevalence of PIM using the three explicit PIM criteria, variations in the most commonly prescribed PIM were observed. For instance, the MALPIP identified diuretics and beta blockers as the top three most commonly prescribed PIM classes, which had lower prevalence in the STOPP and Beers criteria; while the STOPP and Beers criteria labelled antihistamines and opiate analgesics as the second most commonly prescribed PIM classes, respectively. Nevertheless, all criteria identified the pervasive use of PPIs and NSAIDs, consistent with findings from recent studies (Ben-Eltriki et al., 2024; Rodrigues et al., 2024; Wongrakpanich et al., 2018). While NSAIDs are frequently prescribed for age-related pain conditions, PPIs are often co-prescribed for their perceived gastro-protective benefits (Gwee et al., 2018). Additionally, benzodiazepines have been identified as one of the most common PIMs in several studies (Lukačišinová et al., 2024; Rodrigues et al., 2024; Tian et al., 2023), with high detection rates in this study using STOPP and Beers criteria. However, the MALPIP criteria did not highlight benzodiazepines as prominently, due to its higher sensitivity towards a broader class of medications.

The cost analysis outlined varying degrees of cost savings associated with different PIM criteria. The MALPIP criteria, being the most sensitive, offered the highest potential cost savings, reaching up to MYR 45 (approximately USD 10) per month per patient. In contrast, the lowest savings scenario was observed when using the STOPP criteria, with a 25% reduction in PIMs resulting in a cost saving of MYR 4.83 (approximately USD 1) per month. These findings are consistent with a deprescribing trial among frail older adults in Australia (Okafor et al., 2024), which reported annual savings from deprescribed medications ranging from USD 109 to USD 218, translating to USD 9 to USD 18 per month, aligning with our best-case estimates. These findings suggest that the cost savings of deprescribing interventions vary across different medication groups and populations, warranting further cost-effectiveness research to understand these dynamics comprehensively.

The comparative analysis of the three explicit PIM criteria reveals notable differences in their predictive capabilities for adverse outcomes such as emergency department (ED) visits, readmissions, and mortality. The STOPP criteria demonstrated the lowest sensitivity and highest specificity, indicating an accurate but limited ability to identify patients at risk for these adverse events. In contrast, the Beers criteria exhibited higher sensitivity but lower specificity across all outcomes, suggesting that while they are better at detecting at-risk patients, but also result in more false positives. The specificity and sensitivity of the STOPP and Beers criteria in detecting emergency department visits and hospitalisations were found to be comparatively higher in another study conducted in the United States (Brown et al., 2016). This discrepancy could be attributed to the use of older versions of the Beers and STOPP criteria in that study, as well as differences in the study population.

The MALPIP criteria, however, showed exceptionally high sensitivity for predicting ED visits, readmissions, and mortality, but at the expense of very low specificity. This high sensitivity implies that the MALPIP criteria are highly effective at detecting patients who may experience adverse outcomes, but the low specificity indicates a high rate of false positives, which could lead to over-identification and unnecessary interventions. The specificity of the MALPIP criteria could be improved by incorporating patient-specific factors such as gender, comorbidities, clinical parameters, and functional status, like the approach used in STOPP criteria (O’Mahony et al., 2015).

We observed a poor concordance between the three criteria. This can be attributed to differences in their classification structures and development processes. The Beers and MALPIP criteria classify PIMs based on medication classes, while the STOPP criteria categorise them by physiological systems. Additionally, the MALPIP criteria overlap with the STOPP criteria by 54.7% and the Beers criteria by 38.5%, largely due to differences in drug formularies across countries (Chang et al., 2023). Similar findings were reported by Brown et al., with a kappa agreement of 0.58 between the 2012 Beers and 2008 STOPP criteria (Brown et al., 2016). These variations highlight that no single tool comprehensively captures all PIMs, underscoring the importance of using these criteria complementarily in clinical practice to improve PIM detection.

In the deprescribing case scenarios, geriatricians favoured deprescribing medications not indicated for long-term use, such as NSAIDs, PPIs, and benzodiazepines, as well as anticholinergics, disease-specific PIMs, and hypotensive agents. This preference reflects a prioritisation to mitigate risks where the potential harm of continued use outweighs the benefits. Conversely, they were hesitant to discontinue medications with a high risk of withdrawal or those where discontinuation might lead to relapse, such as antipsychotics, similar to previous literature (Robinson et al., 2024). This cautious approach underscores the need to balance the benefits of deprescribing against the risk of adverse outcomes, highlighting the importance of patient-centred assessments and structured deprescribing guidelines in managing polypharmacy among older adults (Goh et al., 2023; Okeowo et al., 2023). Incorporating insights from geriatricians can enhance the specificity of explicit PIM criteria, ensuring they are more effective and contextually relevant for clinical practice.

This study is not without limitation. The study's reliance on data from tertiary hospitals in specific Malaysian states may limit the generalizability of the findings to other settings or countries, and the data retrieved from outpatient hospital setting may not reflect the prescribing patterns and PIM prevalence in primary care or other settings. Future research should consider evaluating cost-effectiveness of deprescribing interventions, examining the real-life applicability of explicit deprescribing criteria and improving the specificity of these tools.

## Conclusion

This study highlights the significant variability in the detection of PIMs among older adults using the MALPIP, Beers, and STOPP criteria, with MALPIP demonstrating the highest sensitivity but the lowest specificity. The findings underscore the importance of context-specific assessments and the role of clinical judgment in optimising medication safety and efficacy in geriatric pharmacotherapy. Further research is needed to explore the cost-effectiveness of deprescribing interventions and to refine explicit PIM criteria to better predict clinical outcomes and balance the benefits and risks of deprescribing among older adults in diverse healthcare settings.

## Supplementary Material

Supplemental Material

## Data Availability

All data to reproduce the tables and figures in the manuscript and Supplemental Material can be obtained with reasonable request from the corresponding author.

## References

[CIT0001] Alhawassi, T. M., Alatawi, W., & Alwhaibi, M. (2019). Prevalence of potentially inappropriate medications use among older adults and risk factors using the 2015 American geriatrics society beers criteria. *BMC Geriatrics*, *19*(1), 1–8. 10.1186/s12877-019-1168-1. 31142286 PMC6542098

[CIT0002] Ang, W. C., Zulkepli, N. S., Mukhtar, N. S., & Zulkefli, N. A. (2021). Prevalence, factors and cost comparison due to potentially inappropriate medications (PIMs) of elderly outpatients in a state hospital in Malaysia. *Journal of Pharmacy*, *1*(1), 27–33. 10.31436/jop.v1i1.54

[CIT0003] Ansah, J. P., & Chiu, C.-T. (2022). Projecting the chronic disease burden among the adult population in the United States using a multi-state population model. *Frontiers in Public Health*, *10*, 1–11. 10.3389/fpubh.2022.1082183. PMC9881650 36711415

[CIT0004] Ben-Eltriki, M., Chhabra, M., Cassels, A., & Wright, J. M. (2024). Inappropriate use of proton pump inhibitor Among elderly patients in British Columbia: What are the long-term adverse events? *Current Drug Safety*, *19*(2), 244–247. 10.2174/1574886318666230726124540 37496243 PMC10788903

[CIT0005] Brown, J. D., Hutchison, L. C., Li, C., Painter, J. T., & Martin, B. C. (2016). Predictive validity of the beers and screening tool of older persons’ potentially inappropriate prescriptions (STOPP) criteria to detect adverse drug events, hospitalizations, and emergency department visits in the United States. *Journal of the American Geriatrics Society*, *64*(1), 22–30. 10.1111/jgs.13884 26782849 PMC5287350

[CIT0006] By the 2019 American Geriatrics Society Beers Criteria® Update Expert Panel. (2019). American geriatrics society 2019 updated AGS beers criteria® for potentially inappropriate medication Use in older adults. *Journal of the American Geriatrics Society*, *67*(4), 674–694. 10.1111/jgs.15767 30693946

[CIT0007] Cateau, D., Ballabeni, P., & Niquille, A. (2021). Effects of an interprofessional quality circle-deprescribing module (QC-DeMo) in Swiss nursing homes: A randomised controlled trial. *BMC Geriatrics*, *21*(1), 1–11. 10.1186/s12877-021-02220-y. 33933030 PMC8088558

[CIT0008] Chang, C.-T., Ang, J.-Y., Islam, M. A., Chan, H.-K., Cheah, W.-K., & Gan, S. H. (2021). Prevalence of drug-related problems and complementary and alternative medicine Use in Malaysia: A systematic review and meta-analysis of 37,249 older adults. *Pharmaceuticals (Basel, Switzerland)*, *14*(3), 1–15. 10.3390/ph14030187. PMC7996557 33669084

[CIT0009] Chang, C. T., Chan, H. K., Cheah, W. K., Tan, M. P., Ch’ng, A. S. H., Thiam, C. N., … Lee, S. W. H. (2023). Development of a Malaysian potentially inappropriate prescribing screening tool in older adults (MALPIP): A Delphi study. *Journal of Pharmaceutical Policy and Practice*, *16*(1), 1–15. 10.1186/s40545-023-00630-4. 37858273 PMC10588247

[CIT0010] Goh, S. S. L., Lai, P. S. M., Ramdzan, S. N., & Tan, K. M. (2023). Weighing the necessities and concerns of deprescribing among older ambulatory patients and primary care trainees: A qualitative study. *BMC Primary Care*, *24*(1), 1–12. 10.1186/s12875-023-02084-8. 37391698 PMC10311750

[CIT0011] Gwee, K. A., Goh, V., Lima, G., & Setia, S. (2018). Coprescribing proton-pump inhibitors with nonsteroidal anti-inflammatory drugs: Risks versus benefits. *Journal of Pain Research*, *11*, 361–374. 10.2147/JPR.S156938 29491719 PMC5817415

[CIT0012] Hacker, K. (2024). The burden of chronic disease. *Mayo Clinic Proceedings. Innovations, Quality & Outcomes*, *8*(1), 112–119. 10.1016/j.mayocpiqo.2023.08.005 PMC10830426 38304166

[CIT0013] Hermann, M., Carstens, N., Kvinge, L., Fjell, A., Wennersberg, M., Folleso, K., Skaug, K., Seiger, A., Cronfalk, B. S., & Bostrom, A.-M. (2021). Polypharmacy and potential drug-drug interactions in home-dwelling older people – A cross-sectional study. *Journal of Multidisciplinary Healthcare*, *14*, 589–597. 10.2147/JMDH.S297423 33727821 PMC7955724

[CIT0014] Jones, C. H., & Dolsten, M. (2024). Healthcare on the brink: Navigating the challenges of an aging society in the United States. *Npj Aging*, *10*(1), 1–10. 10.1038/s41514-024-00148-2. 38582901 PMC10998868

[CIT0015] Kua, C.-H., Yeo, C. Y. Y., Tan, P. C., Char, C. W. T., Tan, C. W. Y., Mak, V., Leong, I. Y.-O., & Lee, S. W. H. (2021). Association of deprescribing With reduction in mortality and hospitalization: A pragmatic stepped-wedge cluster-randomized controlled trial. *Journal of the American Medical Directors Association*, *22*(1), 82–89. 10.1016/j.jamda.2020.03.012 32423694

[CIT0016] Lukačišinová, A., Reissigová, J., Ortner-Hadžiabdić, M., Brkic, J., Okuyan, B., Volmer, D., … Fialová, D. (2024). Prevalence, country-specific prescribing patterns and determinants of benzodiazepine use in community-residing older adults in 7 European countries. *BMC Geriatrics*, *24*(1), 1–13. 10.1186/s12877-024-04742-7. 38454372 PMC10921596

[CIT0017] Okafor, C. E., Keramat, S. A., Comans, T., Page, A. T., Potter, K., Hilmer, S. N., Lindley, R. I., Mangin, D., Naganathan, V., & Etherton-Beer, C. (2024). Cost-Consequence analysis of deprescribing to optimize health outcomes for frail older people: A within-trial analysis. *Journal of the American Medical Directors Association*, *25*(3), 539–544.e2. 10.1016/j.jamda.2023.12.016 38307120

[CIT0018] Okeowo, D. A., Zaidi, S. T. R., Fylan, B., & Alldred, D. P. (2023). Barriers and facilitators of implementing proactive deprescribing within primary care: A systematic review. *The International Journal of Pharmacy Practice*, *31*(2), 126–152. 10.1093/ijpp/riad001 36860190

[CIT0019] O’Mahony, D., O’Sullivan, D., Byrne, S., O’Connor, M. N., Ryan, C., & Gallagher, P. (2015). STOPP/START criteria for potentially inappropriate prescribing in older people: Version 2. *Age and Ageing*, *44*(2), 213–218. 10.1093/ageing/afu145 25324330 PMC4339726

[CIT0020] Omuya, H., Nickel, C., Wilson, P., & Chewning, B. (2023). A systematic review of randomised-controlled trials on deprescribing outcomes in older adults with polypharmacy. *International Journal of Pharmacy Practice*, *31*(4), 349–368. 10.1093/ijpp/riad025 37155330

[CIT0021] Osanlou, R., Walker, L., Hughes, D. A., Burnside, G., & Pirmohamed, M. (2022). Adverse drug reactions, multimorbidity and polypharmacy: A prospective analysis of 1 month of medical admissions. *BMJ Open*, *12*(7), 1–7. 10.1136/bmjopen-2021-055551. PMC9255409 35788071

[CIT0022] Robinson, M., Mokrzecki, S., & Mallett, A. J. (2024). Attitudes and barriers towards deprescribing in older patients experiencing polypharmacy: A narrative review. *Npj Aging*, *10*(1), 1–6. 10.1038/s41514-023-00132-2. 38263176 PMC10806180

[CIT0023] Rodrigues, D. A., Herdeiro, M. T., Mateos-Campos, R., Figueiras, A., & Roque, F. (2024). Comparing AGS beers 2019, STOPP version 2, and EU(7)-PIM list in Portuguese older adults in primary health care. *European Journal of Clinical Pharmacology*, *80*(4), 603–612. 10.1007/s00228-024-03633-5 38319349 PMC10937751

[CIT0024] S. AlHarkan, K., Alsousi, S., AlMishqab, M., Alawami, M., Almearaj, J., Alhashim, H., … AlOmar, R. S. (2023). Associations between polypharmacy and potentially inappropriate medications with risk of falls among the elderly in Saudi arabia. *BMC Geriatrics*, *23*(1), 1–9. 10.1186/s12877-023-03852-y. 37024805 PMC10080807

[CIT0025] Tian, F., Chen, Z., Zeng, Y., Feng, Q., & Chen, X. (2023). Prevalence of use of potentially inappropriate medications among older adults worldwide: A systematic review and meta-analysis. *JAMA Network Open*, *6*(8), 1–15. 10.1001/jamanetworkopen.2023.26910. PMC10398411 37531105

[CIT0026] Varghese, D., Ishida, C., Patel, P., & Haseer Koya, H. (2024). Polypharmacy. In *StatPearls*. StatPearls Publishing. http://www.ncbi.nlm.nih.gov/books/NBK532953/. 30422548

[CIT0027] Wongrakpanich, S., Wongrakpanich, A., Melhado, K., & Rangaswami, J. (2018). A comprehensive review of non-steroidal anti-inflammatory drug use in the elderly. *Aging and Disease*, *9*(1), 143–150. 10.14336/AD.2017.0306 29392089 PMC5772852

