# Supplementary material for: Comparing Beers, STOPP and MALPIP criteria in detecting potentially inappropriate medications, clinical outcomes, and cost impacts among older Malaysians: a cohort study
Source: J Pharm Policy Pract. 2024 Dec 18;17(1):2436896. doi: 10.1080/20523211.2024.2436896 (PMC11809192; doi:10.1080/20523211.2024.2436896)
Supplement: Supplemental Material [file JPPP_A_2436896_SM0072.docx]

**Supplementary Table 1: Predictive factors of emergency department visits using univariate and multivariate logistic regression**

|  | **Unadjusted odd ratios (95% CI)** | **p-value** | **Adjusted odd ratios (95% CI)** | **p-value** |
| --- | --- | --- | --- | --- |
| Age  60-70  71-80  81 and above | Reference  0.67 (0.49-0.90)  0.84 (0.53-1.34) | 0.007  0.465 | 0.68 (0.50-0.93)  0.82 (0.51-1.33) | 0.014  0.425 |
| Gender  Male  Female | Reference  0.90 (0.68-1.12) | 0.446 |  |  |
| Hospitalization past 12 months  No  Yes | Reference  0.35 (0.26-0.48) | <0.001 | 0.38 (0.28-0.52) | <0.001 |
| Total meds | 0.92 (0.88-0.95) | <0.001 | 0.94 (0.90-0.98) | 0.008 |
| Total comorbid | 0.79 (0.71-0.89) | <0.001 | 0.86 (0.77-0.97) | 0.016 |
| Beers criteria  No  Yes | Reference  0.86 (0.65-1.13) | 0.280 |  |  |
| STOPP criteria  No  Yes | Reference  1.10 (0.82-1.47) | 0.530 |  |  |
| MALPIP  No  Yes | Reference  1.21 (0.79-1.86) | 0.379 |  |  |

**Supplementary Table 2: Predictive factors of hospital readmission visits using univariate and multivariate logistic regression**

|  | **Unadjusted odd ratios (95% CI)** | **p-value** | **Adjusted odd ratios (95% CI)** | **p-value** |
| --- | --- | --- | --- | --- |
| Age  60-70  71-80  81 and above | Reference  1.39 (1.03-1.87)  0.99 (0.62-1.59) | 0.030  0.969 |  |  |
| Gender  Male  Female | Reference  0.89 (0.67-1.17) | 0.405 |  |  |
| Hospitalization past 12 months  No  Yes | Reference  0.27 (0.19-0.36) | <0.001 | 3.54 (2.59-4.86) | <0.001 |
| Total meds | 1.10 (10.5-1.14) | <0.001 | 1.07 (1.02-1.12) | 0.004 |
| Total comorbid | 1.30 (1.16-1.44) | <0.001 | 1.17 (1.03-1.32) | 0.014 |
| STOPP criteria  No  Yes | Reference  1.51 (1.12-2.04) | 0.007 | 1.53 (1.12-2.10) | 0.008 |
| Beers criteria  No  Yes | Reference  0.91 (0.69-1.20) | 0.508 |  |  |
| MALPIP  No  Yes | Reference  0.82 (0.53-1.25) | 0.352 |  |  |

**Supplementary Table 3: Predictive factors of mortality using univariate and multivariate logistic regression**

|  | **Unadjusted odd ratios (95% CI)** | **p-value** | **Adjusted odd ratios (95% CI)** | **p-value** |
| --- | --- | --- | --- | --- |
| Age  60-70  71-80  81 and above | Reference  1.68 (1.10-2.55)  1.85 (1.03-3.34) | 0.015  0.041 | 1.63 (1.07-2.49)  1.90 (1.05-3.45) | 0.022  0.035 |
| Gender  Male  Female | Reference  1.12 (0.76-1.65) | 0.562 |  |  |
| Hospitalization past 12 months  No  Yes | Reference  2.19 (1.46-3.30) | <0.001 | 2.09 (1.38-3.16) | <0.001 |
| Total meds | 0.98 (0.92-1.04) | 0.470 |  |  |
| Total comorbid | 1.21 (1.04-1.40) | 0.013 | 1.17 (1.00-1.36) | 0.040 |
| STOPP criteria  No  Yes | Reference  1.13 (0.77-1.69) | 0.523 |  |  |
| Beers criteria  No  Yes | Reference  1.29 (0.87-1.90) | 0.199 |  |  |
| MALPIP  No  Yes | Reference  1.52 (0.75-3.09) | 0.248 |  |  |
